# Decreased Glasgow Coma Scale score in medical patients as an indicator for intubation in the Emergency Department: Why are we doing it?

**DOI:** 10.6061/clinics/2021/e2282

**Published:** 2021-03-01

**Authors:** Sabrina Correa da Costa Ribeiro

**Affiliations:** Emergencias Clinicas, Faculdade de Medicina FMUSP, Universidade de Sao Paulo, Sao Paulo, SP, BR

## BACKGROUND

“My grandfather was intubated yesterday. I am not comfortable with this. I mean, he is 101 years old!”

“Do you know what happened?”

“They did not tell me exactly. They said they got worried because he was drowsy and not very responsive.”

Her grandfather, Mr. B, had never completed an advance directive. He was still lucid and had been relatively active until the preceding two months. His daughter was concerned and brought him to the emergency department (ED) because in the last week he appeared weak and had stopped eating and talking.

Within hours of arrival, Mr. B was intubated and transferred to the intensive care unit (ICU). No one had asked about his wishes. His daughter never thought a simple visit to the ED would spiral into that. As the doctor on call explained:

“He was just fine until a few months ago. We had to do everything.”

##  INTRODUCTION

Mr. B died a few hours later. According to a recently published paper that studied over 30,000 patients aged >65 years who were intubated in the ED (1), those past the age of 90 years, like Mr. B, had a 50% chance of dying in the hospital and a 36% chance of being discharged to a nursing home. (His granddaughter later informed me that Mr. B would never have desired or accepted either outcome.) The indication for intubating Mr. B was decreased consciousness and unresponsiveness. In the same study (1), altered mental status or seizures was found to account for 15% of all intubations, representing a total of 5,259 patients.

Some might surmise that intubating a 101-year-old patient is unethical or nonsensical. However, basing this decision solely on a patient’s age might be considered inappropriate and even ageist. A “do not intubate” or “do not resuscitate” status must not be assumed, and a patient’s age alone is not an indication to withhold care in acute illness. Studies suggest that clinicians caring for older adults at the end of life might underestimate the level of resuscitation desired by the patient (2). The focus of this review is not to state that older patients should not be intubated. Instead, we intend to discuss the lack of evidence behind using decreased consciousness as a sole indicator of intubation.

The Glasgow Coma Scale (GCS) was originally developed for trauma patients (3) as a means of enabling standardized evaluation and recording a patient’s level of consciousness. Established trauma guidelines (4) recommend early intubation for patients with a GCS score ≤8, according to the findings from early studies (5,6), which showed a lower rate of complications with early airway protection. This approach has also been widely used for medical patients, although there are no prospective, controlled studies that validate this strategy in a non-trauma population. Some authors recommend placing an advanced airway in all patients with a GCS score of 8 or lower (7,8), whereas others suggest a more individualized approach (9,10). The aim of this study is to review the literature on decreased consciousness, airway reflexes, and intubation in medical patients and reflect on their potential harms and benefits.

## GLASGOW COMA SCORE: RELIABILITY AND ASSOCIATION WITH AIRWAY REFLEXES

Decreased consciousness is a common reason for presentation to the ED and one of the main reasons for endotracheal intubation. The most common causes of acute brain injury are traumatic, ischemic/hemorrhagic, and metabolic (drugs, excess insulin, diabetes, and alcohol) (8). GCS is a widely known and used tool for mental status assessment and is believed to be both reproducible and reliable. However, in one study of independent paired assessments by attending emergency physicians, GCS scores were found to be the same in just 38% of patients and were 2 or more points apart in 33% of patients (11). The same GCS score may also correspond to multiple permutations of its constituent elements, holding very different significance for the same absolute number. Healey reported that the mortality rate associated with three different constituent elements of the GCS score of four laypeople was between 19% and 48%, where utilization of the motor component of the score alone revealed a better correlation with survival (12).

Another controversial issue is how likely a patient is to lose the gag reflex if the GCS score drops. A study by Rotheray et al. (13) showed that gag and cough reflexes decrease with a decreasing GCS score in patients requiring critical care, but there was no evidence of a sudden drop in these reflexes at a GCS score of 8. Among those with GCS scores of 9-14, the authors found absent reflexes in 37%, and among those with a GCS score of 8 or lower, 63% showed the absence of a gag reflex. Furthermore, 22% of those with a GCS score of 15 showed no gag reflex, revealing that although the GCS score can be taken into consideration, it is far from perfect as a predictor of protective airway reflexes.

## WHAT IS THE POTENTIAL FOR HARM IF WE DO NOT INTUBATE THESE PATIENTS?

Unfortunately, there is no randomized controlled trial showing the risk/benefit profile of intubation *versus* a conservative approach in medical patients with a low GCS score. In a small ED study (14), Duncan et al. described a prospective cohort of 73 patients with decreased GCS scores (ranging from 3 to 14) as a result of drug or alcohol intoxication. Only one patient (who had a GCS score of 12) required intubation. None of the patients with a GCS score of 8 or less aspirated or required intubation. A retrospective study by Van Helmond showed a low rate of major complications and the need for intubation in a cohort of 209 intoxicated patients admitted to the ED (15). In older patients, such as those with hypoactive delirium, the reliability of the GCS score might be even lower because this scale was not validated in older patients and because baseline GCS scores can be chronically abnormal in patients with dementia. Accordingly, the use of the GCS in this population has been questioned (16), and modifications of the verbal component for suitability to a patient’s baseline status have been suggested.

## WHAT IS THE POTENTIAL FOR HARM IF WE DO INTUBATE THESE PATIENTS?

An article by Rubin et al. (17) described situations considered worse than death by 180 hospitalized patients with serious illnesses. Of these patients, 67% considered requiring a breathing tube a state worse than death. In the Introduction section of this article, we report a high mortality (1) of elderly patients intubated in the ED. A recent study also questioned the dogma of intubating patients with a GCS score lower than 8, showing that intubation at arrival was associated with an increase in mortality and longer ICU and overall length of stay (18).

Forte et al. (19) suggests a bioethical framework to guide decision making that relies on four steps: evidence-based practice, comprehension of biography and values, situational awareness/clinical team judgment, and ethics of deliberation.

Applying the concepts of this framework to intubation based solely on GCS scores, we conclude the following: 1) this practice is not evidence-based; 2) it is applied in a setting in which, in most cases, patients’ wishes are difficult to assess; and 3) this intervention has a low likelihood of yielding the desired outcome (total or partial recovery with preservation of cognitive and other functions) and a significant possibility of resulting in an outcome that most patients would find undesirable (death or life on a breathing tube).

## CONCLUSION

Although it is a widespread practice, there is no evidence to support intubation of medical patients solely on the basis of a GCS score less than 8. Other factors, such as disease trajectory, acute diagnosis, and prognosis, must be considered, and it is probably safe to delay the decision to intubate until a family member arrives or records are obtained to give the clinician more information on the patient’s values and preferences.
